# Medicinal plants for gingivitis: a review of clinical trials

**DOI:** 10.22038/IJBMS.2018.31997.7690

**Published:** 2018-10

**Authors:** Hannaneh Safiaghdam, Vahideh Oveissi, Roodabeh Bahramsoltani, Mohammad Hosein Farzaei, Roja Rahimi

**Affiliations:** 1 Student Research Committee, Dental School, Shahid Beheshti University of Medical Sciences, Tehran, Iran; 2 PhytoPharmacology Interest Group (PPIG), Universal Scientific Education and Research Network (USERN), Tehran, Iran; 3 Faculty of Pharmacy, Tehran University of Medical Sciences, Tehran, Iran; 4 Department of Pharmacy in Persian Medicine, School of Persian Medicine, Tehran University of Medical Sciences, Tehran, Iran; 5 Pharmaceutical Sciences Research Center, Kermanshah University of Medical Sciences, Kermanshah, Iran; 6 Medical Biology Research Center, Kermanshah University of Medical Sciences, Kermanshah, Iran

**Keywords:** Gingivitis, Medicinal plants, Oral diseases, Phytochemicals, Traditional medicine

## Abstract

**Objective(s):**

Periodontal diseases are among prevalent oral health problems which may ultimately lead to severe complications in oral cavity. Herbal products can be designed as single or multicomponent preparations for better oral health. This study aims to review current clinical trials on the effectiveness of herbal products in gingivitis.

**Materials and Methods:**

Electronic databases, including PubMed, Scopus, ScienceDirect and Cochrane library were searched with the keywords “gingivitis” in the title/abstract and “plant/ extract/ herb” in the whole text for clinical trials on herbal treatments for gingivitis. Data were collected from 2000 until January 2018. Only papers with English full-texts were included in our study.

**Results:**

Herbal medicines in the form of dentifrice, mouth rinse, gel, and gum were assessed in gingivitis via specific indices including plaque index, bleeding index, microbial count, and biomarkers of inflammation. Pomegranate, aloe, green tea, and miswak have a large body of evidence supporting their effectiveness in gingivitis. They could act via several mechanisms such as decrease in gingival inflammation and bleeding, inhibition of dental plaque formation, and improvement in different indices of oral hygiene. Some polyherbal formulations such as triphala were also significantly effective in managing gingivitis complications.

**Conclusion:**

Our study supports the efficacy and safety of several medicinal plants for gingivitis; however, some plants do not have enough evidence due to the few number of clinical trials. Thus, future studies are mandatory for further confirmation of the efficacy of these medicinal plants.

## Introduction

Periodontal diseases, including gingivitis and periodontitis, are amongst the prevalent oral health problems which may ultimately lead to severe conditions in oral cavity (1). Gingivitis is the inflammation of gingiva without apical migration of junctional epithelium which, unless treated, will lead to periodontitis in susceptible patients (1, 2). Gingivitis has a high prevalence among societies. In an epidemiological study in American adults, nearly 55.7% of subjects had a GI index (Löe-Silness Gingivitis Index) higher than 1 (3). Various etiological factors have been introduced regarding periodontal diseases since it is considered a multifactorial disease (4). Biofilm accumulation and pathogens are the key contributors; however, other risk factors can be categorized as modifiable factors such as smoking, obesity, stress, diabetes mellitus, osteoporosis, and Vitamin D and calcium deficiency, as well as non-modifiable factors like genetic polymorphisms (5). It has been shown that there is a negative correlation between gingivitis and oral-health related quality of life (6). 

Mechanical removal of plaque via tooth brush and use of dental floss has been considered as an effective method in controlling gingivitis (7). Nevertheless, adequate time of brushing, efficient cleaning of all tooth surfaces and regular oral hygiene is hard to achieve in every individual due to variations in oral health practices which accounts for high prevalence of gingivitis (8). Therefore, additional approaches such as dentifrices and mouthwashes containing chemical or herbal agents are suggested (9). American Dental Association has approved chlorhexidine (CHX) and essential oils (EO) as antiseptics in mouthwashes (10); though, there have been reports of hypersensitivity, stain formation on teeth surface, oral mucosa irritation, and altered taste with chlorhexidine (10, 11). 

Phytotherapy in oral health has received attention lately and a plethora of clinical trials have been conducted in this area (12-16). Herbs are known to have anti-inflammatory, antimicrobial and antioxidative effects (17). Herbal products in the forms of dentifrices and mouth rinses can be based on a single natural component, or a mixture of several medicinal plants (18). The aim of this study is to comprehensively review literature and provide an overview upon effectiveness, safety and availability of herbal products for gingivitis.

## Materials and Methods

Electronic databases, including PubMed, Scopus, ScienceDirect and Cochrane library were searched for clinical trials on herbal treatments for gingivitis. The following keywords were used: Gingivitis (title/abstract) AND plant/extract/herb (all fields). We searched for articles in English from 2000 until January 2018 and checked their reference list for additional relevant studies. A total of 1998 articles were collected. Total of 883 duplicate results were excluded. Abstracts and titles were screened and 898 articles were excluded as they were *in vitro* studies or investigating oral diseases other than gingivitis. Studies on mixtures of chemical and herbal components were also excluded because the pharmacological activity could not be completely attributed to the herbal component. The number of 34 articles were excluded because they were reviews. Nine articles were excluded since the full-texts were not in English. A total of 60 relevant articles were published before 2000 which were excluded as we intend to focus on recent trends. Full-texts for the remainder were obtained. Ten articles were excluded as they were about non-herbal materials (animal and fungal origin). 

The included studies were screened for scientific names of herbal agents, their concentrations and types of preparation, duration of study, tests and indices used to evaluate the outcome and characteristics of subjects. Jadad score was used to compare the methodology of the included articles (19). Outcomes were compared between the herbal component and positive/ placebo controls. In case of before-and-after studies, baseline and final records were compared. The arrows (↑ and ↓) show increase and decrease in the specified parameter, respectively. 

## Results

***Single herbal preparations***

*Aloe vera (Aloe)*

*Aloe vera* (L.) Burm. f. (synonym: *Aloe barbadensis*) or Aloe from the family Asphodelaceae (Liliaceae) (20) is a perennial plant which originates from South Africa, but has also been cultivated in dry subtropical and tropical regions, such as the southern USA (21, 22).

Potentially active compounds of the leaves include water- and fat-soluble vitamins, simple/complex polysaccharides, minerals, organic acids, and phenolic compounds (22).

In a double-blind, randomized clinical study on 45 subjects, daily rinse with 15 ml of aloe solution significantly decreased Gingival index (GI) and Sulcus bleeding index (SBI) after three months. GI, describes the severity of gingivitis (23); while SBI is an index of gingival inflammation in which bleeding is measured from four gingival units (24). The reduction was more pronounced when scaling and root planning was added to this treatment (25). Another study also demonstrated that aloe can be used as an adjunct to scaling to improve clinical parameters such as PI, GI and bleeding on probing (BOP) (26). Plaque index (PI), developed by Silness and Loe in 1964, assesses the thickness of the plaque in the margin of the tooth closest to gums (23). BOP is the earliest clinical symptom of gingivitis and a predictor of periodontal stability described by Lang *et al* (27). In another study on 120 subjects, aloe 100% solution consumed for 7 days was effective in reducing PI, bleeding index (BI), and modified gingival index (MGI), a score introduced by Lobene in 1985 to assess the severity of gingivitis by non-invasive approaches (28); however, the effectiveness was less than chlorhexidine 2% (CHX) (29). In a clinical trial on 30 subjects, aloe dentifrice showed an efficacy similar to fluoridated dentifrice after 30 days of brushing as they equally reduced PI and gingival bleeding index (GBI) (30). Same results were obtained in a study on 345 subjects who were advised to rinse with aloe or CHX for 30 days (31). In another controlled study, aloe dentifrice was proved equally effective as a control commercial product (Sensodyne) in improving GI and PI indices (32). By contrast, the effect of aloe mouth rinse on PI and GI was compared with CHX and chlorine dioxide in a study for 15 days in which aloe had a significantly lower efficacy (33). The obtained result may be due to the shorter treatment period in comparison to previous studies. Other parameters such as Quigley-Hein plaque index (QHI a modification of PI that evaluates the plaque revealed on the buccal and lingual non-restored surfaces of the teeth (34) and microbial count was significantly reduced in a study on 90 subjects with both aloe and a triclosan containing fluoride dentifrice compared to placebo (35). 

*Azadirachta indica (Neem)*

*Azadirachta indica* A. Juss or Neem from the family Meliaceae is a tree which is mainly cultivated in the Indian subcontinent (36). The main active components of the plant with important antibacterial activity are nimbidin, nimbinin, and azadirachtin (37). 

In a randomized clinical trial on 30 subjects, neem mouthwash was compared to *Camellia sinensis* (tea) and CHX mouthwash. Both herbal extracts improved GI, PI, OHIS (a simplified version of the OHI which combines the Debris Index and the Calculus Index on 6 tooth surfaces) (38) and pH level better than CHX; however, green tea outperformed neem in PI index (39). In another study, rinsing with neem and CHX mouthwash reduced PI, SBI and GI indices after 4 weeks with no significant difference between the two agents (40). Sharma *et al.* compared neem mouthwash with mango and CHX. Neem and CHX had similar results in reducing PI and GI but CHX had a more sustained effect after one month (41). Another parameter used to evaluate the effect of neem was interleukin-2 (IL-2) and interferon-γ (IFN-γ) levels. Results showed that the reduction of PI, GI, and IL-2 and IFN-γ level with CHX, essential oil, and povidone iodine is statistically more significant than neem (42).

*Calendula officinalis (Marigold)*

*Calendula officinalis* L. or marigold from the family Asteraceae (Compositae) is a plant native to Central and Southern Europe, Western Asia and the US, but it is widely cultivated as an ornamental plant in other parts of the world. Whole plant contains terpenoids, quinones, flavonoids, coumarines, volatile oil, and carotenoids (43, 44).

A study involving 240 subjects showed that marigold mouthwash can significantly improve GI, PI, SBI and OHIS indices after 3 months of treatment (45). In another clinical trial without a control group, marigold dentifrice reduced GI, PI and BOP in 40 patients with stablished gingivitis (46). 

*Camellia sinensis (Green tea)*

*Camellia sinensis* (L.) Kuntze or tea from the family Theaceae is an evergreen plant originating from China which later spread to other parts of the world. The major chemical components of tea are polyphenols like catechins and flavonoids, as well as methylxanthine alkaloids including caffeine, theobromine, and theophylline. Based on the process, several types of tea are produced amongst which the most popular ones are green tea, as the unfermented type which mostly contains catechin derivatives, and black tea, with the highest degree of fermentation in which the major polyphenols are theaflavins (47).

In a clinical study, green tea improved GI, PI, OHIS and pH level better than CHX or neem (39). PI was equally improved using either green tea or CHX mouthwash in a clinical trial on 30 subjects (48). PI and GI indices were decreased in 110 subjects after using green tea mouthwash for a month (49). Hydroxypropylcellulose strips were used as a sustained release delivery system in a clinical trial on 6 subjects with advanced periodontitis. Combination of green tea and scaling could reduce pocket probing depth (PPD, the distance from the free gingival margin to the bottom of the pocket or gingival sulcus) (50) and peptidase activity after 8 weeks. Green tea also showed *in vitro* bactericidal activity against *Porphyromonas gingivalis*, *Prevotella intermedia*, *Prevotella nigrescens*, and black-pigmented Gram-negative anaerobic rods (51). However, in a study on subjects with chronic gingivitis, green tea had no significant effect on PI, GI and papillary bleeding index (PBI, a score based on sweeping a probe in the sulcus from the line angle to the interproximal contact (52)) (53). Chew candies containing green tea were also effective in reducing SBI and approximal plaque index (API, another periodontal measure defined to further encourage oral hygiene among patients (54)) compared to placebo (55). Green tea gel also improved periodontal health in 49 patients with chronic gingivitis according to GI and PBI parameters compared to placebo control. GI reduction was more pronounced in CHX while PBI was more reduced with green tea; however, plaque scoring system (PSS, modified form of PI) was not improved by the herbal gel (56). Green tea mouthwash performed equally well compared to CHX according to QHI and GI indices as well tooth and tongue stain parameters. Test treatment improved GBI more than CHX (57). 

*Curcuma longa (Turmeric)*

*Curcuma longa* L. or turmeric from the family Zingiberaceae is a plant native to tropical and subtropical climates, widely cultivated in Asian countries including China and India (58). The main components present in the rhizome are curcuminoids (curcumin, methoxycurcumin, and bisdemethoxycurcumin), as well as the essential oil compounds including turmerones (59, 60).

In a clinical trial, curcumin gel was compared to CHX and a combination of CHX and metronidazole gels. Curcumin was more efficient in reducing PI, MGI, BOP, PPD and IL-1β and CCL28 levels in gingival crevicular fluid (61). In another study, curcumin mouthwash reduced GI and total microbial count to the same level as CHX, and QHI less than CHX (62). Also, in 10 subjects with severe gingivitis, curcumin gel reduced PBI and GI after 3 weeks (63). A combination of turmeric and eugenol resulted in same PI, GI and BAPNA (a method to analyze trypsin like activity of “red” complex microorganisms) values as CHX mouthwash (64). 

*Lippia sidoides (pepper-rosmarin)*

*Lippia sidoides* Cham. or pepper-rosmarin from the family Verbenaceae is a plant which is distributed mostly in Brazil. The leaves contain essential oil with limonene, β-caryophyllene, *p*-cymene, camphor, linalool, α-pinene and thymol as major components (65).

In a double-blind, placebo-controlled clinical study in 22 subjects, pepper-rosmarin gel failed to reduce GBI or PI in comparison to control; however, GI was significantly improved (66). In another study, PI and GBI scores were improved after rinsing with either pepper-rosmarin or CHX gel (13). Effect of pepper-rosmarin mouthwash on PI, GI and GBI indices were assessed in a study involving 55 subjects which showed a similar efficacy to CHX. Salivary *Streptococcus mutans* count was also reduced with both treatments (67, 68).

*Magnolia officinalis (Magnolia)*

*Magnolia officinalis* L. or magnolia is an endangered deciduous tree from the family Magnoliaceae. Due to the medicinal importance, the tree has been over-harvested to obtain its valuable bark. Magnolol and honokiol with lignan structure are the major phenolic constituents of *M. officinalis* bark (69, 70).

In a study on 94 subjects, magnolia mouthwash significantly reduced QHI and GI compared to placebo (16). Magnolia and xylitol chewing gum also improved plaque pH, BOP and reduced salivary *Streptococcus mutans* count after 30 days of treatment (71).

***Matricaria chamomilla*** **(Chamomile)**

*Matricaria* *chamomilla* L. from the family Asteraceae is an annual plant native to eastern and southern parts of Europe; but is also cultivated in several other parts of the world. Numerous phytochemical constituents have been identified in chamomile flower amongst which the most important ones are apigenin, α-bisabolol and cyclic ethers, umbelliferone, and chamazulene (72).

A mouthwash prepared with chamomile extract was as efficient as CHX in reducing visible plaque index (VPI, an index for plaque accumulation and oral hygiene (73)) and GBI (74). Also, in another trial, chamomile mouthwash was compared to pomegranate and miswak mouthwashes in which all herbal treatments could significantly reduce PI and BOP (75).

*Ocimum spp. (Basil)*

*Ocimum* spp. or basil belongs to plant family Lamiaceae (Labiatae). The genus *Ocimum* has around 30 species native to Africa, Asia, and tropical parts of South America (Brazil). The volatile oil of the leaves contains eugenol and methyl eugenol, carvacrol and a sesquiterpine hydrocarbon, caryophyllene. Fresh leaves and stem extract yield some phenolic compounds such as circimaritin, cirsilineol, isothymusin, rosmarinic acid and apigenin which represented antioxidant activity (76, 77).

*Ocimum gratissimum* reduced GBI and PI to same levels as CHX after 3 months in 30 subjects with gingivitis (78). *Ocimum sanctum* also reduced GI and PI to same levels as CHX after one month of treatment in 108 subjects (79).

*Punica granatum (Pomegranate)*

*Punica granatum* L. or pomegranate from the family Lythraceae, is a tree native to Iran, but is now cultivated in some other countries. Both fruit peel and root cortex are used as medicinal parts which contain ellagic acid, ellagitannins (including punicalagins), punicic acid, flavonoids, anthocyanidins, anthocyanins, and estrogenic flavonols and flavones as well as alkaloid like pelletierine (80-82)

In a short-term study, pomegranate mouthwash enhanced GI index after 4 days better than CHX (83). The effect of pomegranate mouthwash on gingivitis was assessed in a clinical study considering total saliva protein (which correlates with amount of plaque forming bacteria), activity level of aspartate aminotransferase (an indicator of cell injury), α-glucosidase activity (a sucrose degrading enzyme), activity level of the antioxidant enzyme ceruloplasmin, and radical scavenging capacity. All the aforementioned parameters were significantly improved after 4 weeks of treatment (84). In another study, pomegranate mouthwash decreased the streptococci count of saliva, but failed to reduce PI and GBI (though to a lesser extent than CHX) (85). In a trial by Salgado *et al.* pomegranate gel showed no significant effect on GBI and PI, either (86). By contrast, pomegranate rinse in patients with diabetes mellitus and gingivitis could reduce GBI, PPD, PI and MGI with an efficacy equal to CHX (87). In addition, pomegranate gel accompanied by mechanical debridement reduced PI, GI, PBI and gram-negative bacilli and cocci count (88). Also, pomegranate mouthwash showed similar efficacy to Persica mouthwash (with *Salvadora persica* as the main ingredient) or Matrica (containing chamomile as the chief active component) regarding PI and BOP indices (75).

*Salvadora persica (Miswak)*

*Salvadora persica* L. or Miswak from the family Salvadoraceae is a medicinal plant with a wide geographic distribution is Asia and Africa. The plant is traditionally used as a natural toothbrush to improve oral health in the native areas. The major components from the essential oil of the tree stem are 1,8-cineole (eucalyptol), β-pinene, α-caryophellene, 9-epi-(E)-caryophellene, and β-sitosterol (89, 90).

In a clinical trial, miswak chewing gum reduced GI and SBI compared to placebo; however, it had no effect on PI. It should be mentioned that several patients complained about the unpleasant taste of the preparation (91). Khalessi *et al.* (2004) also failed to detect a significant improvement in PI by miswak mouthwash; though, GBI and salivary concentrations of *S. mutans* were successfully reduced (92). A dentifrice containing miswak showed effectiveness similar to a commercial product (Parodontax) in reducing SBI and API (93). In another study, colony forming units of plaque samples were similar after using either Persica (a mouthwash containing miswak extract) or Listerine, but the efficacy was less than CHX (94). In another study QHI and GI indices were applied to compare the use of miswak to regular toothbrush. Best results were obtained when both miswak and toothbrush were used (95). Inactivated (boiled) miswak sticks were compared to active sticks in a clinical trial which obtained same results for both preparations with regard to API, GI and sub-gingival microbiota (96)

**Figure 1:**
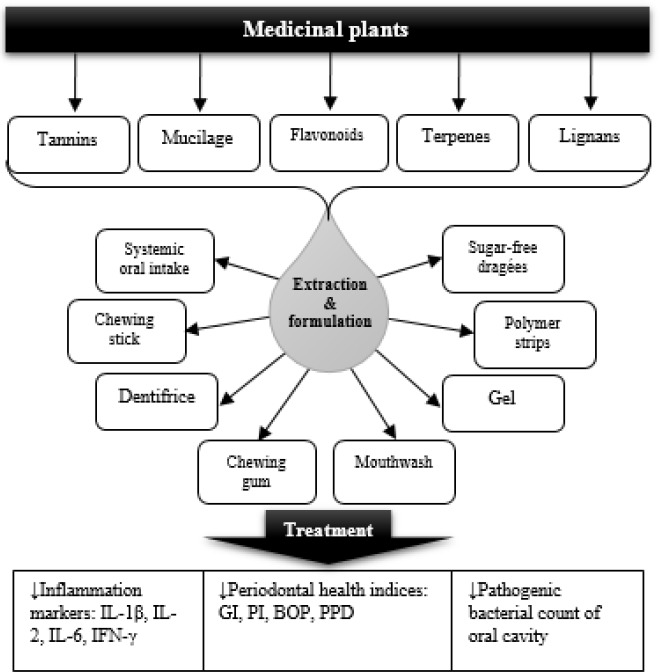
Medicinal plants, active components, formulations, and mechanisms in management of gingivitis. IL: interleukin, IFN: interferon, GI: gingivitis index, PI: plaque index, BOP: bleeding on probing index, PPD: periodontal pocket depth

**Table 1:** Clinical trials on the use of single medicinal plants for the treatment of gingivitis

Plant scientific name	Type of preparation	Study design	Jadad score	Duration of study (day)	Outcomes	Reference
*Acacia arabica*	Dentifrice	Randomized, double-blind, crossover controlled trial in 60 subjects with gingivitis-compared to regular toothpaste	3	28	↓GI, QHI, BOP: better with test	(121)
*Aloe vera*	Dentifrice	Randomized clinical trial in 45 subjects-group 1 (scaling)/ group 2 (scaling + *A. vera*) / group 3 (*A. vera)*	3	42	↓PI, GI, PoB, PPD in all groups with best effect in group 2	(26)
*Aloe vera*	Dentifrice	Randomized, placebo & positively controlled clinical trial in 90 subjects with chronic generalized gingivitis-compared with dentifrice containing fluoride + triclosan	5	168	↓GI, QHI, microbial count same in both groups	(122)
*Aloe vera*	Mouthwash	Single-center, single-blind, controlled trial in 85 subjects- compared to CHX or chlorine dioxide	3	15	↓PI, GI with better effect by CHX and chlorine	(33)
*Aloe vera *100%	Mouthwash	Randomized, double-blind, controlled study in 120 healthy subjects with experimental gingivitis -compared with CHX	2	22	↓BI, MGI, PI in both groups (CHX was better in PI)	(29)
*Aloe vera *15 ml	Mouthwash	Controlled clinical trial in 45 subjects with plaque-induced gingivitis in comparison to scaling only	2	90	↓GI & SBI Better results with *A. vera* mouthwash + scaling	(25)
*Aloe vera* 45%	Dentifrice	Randomized, double-blind, intra-individual & controlled clinical study in 15 subjects with gingivitis-compared with control dentifrice	4	1.5 year	↓PI, GI same in both groups	(32)
*Aloe vera *50%	Dentifrice	Randomized, double-blind, parallel, clinical trial in 30 subjects- compared with fluoridated dentifrice	4	30	↓PI, GBI same in both groups	(30)
*Aloe vera* 99%	Mouthwash	Randomized, triple-blind, controlled trial in 345 subjects- compared with CHX	5	30	↓PI, GI same in both groups	(31)
*Azadirachta indica * 0.01%, Essential oil 0.01%	Mouthwash	Double-blind clinical trial in 80 subjects with gingivitis- compared with CHX and povidone iodine	5	14	↓PI, GI IL-2 & IFN-γ in all groups except *A. indica*	(42)
*Azadirachta indica *0.19%	Mouthwash	Randomized, double-blind, controlled trial in 45 subjects with plaque induced gingivitis-compared with CHX	2	28	↓BI, GI, BOP same in both groups	(40)
*Azadirachta indica *50% OR *Mangifera indica* 50%	Mouthwash	Clinical trial in 97 subjects with gingivitis-compared with CHX	5	21	↓PI Efficacy: CHX=*A. indica*> *M. indica* Duration of effect: CHX= 2 months *A. indica*- *M. indica*: 21 or 28 d ↓GI Efficacy: CHX=*A. indica*> *M. indica* at 21 d or 1 month Duration of effect: CHX= 3 months *A. indica*- *M. indica*: 1 & 2 month	(123)
*Berberis vulgaris *1%	Gel	Double-blind clinical trial in 45 subjects-compared with Colgate anti-plaque dentifrice	1	21	↓PI, GI same in both groups	(118)
*Boswellia serrata *0.1% & 0.2%	Gum	Randomized, double-blind, placebo-controlled trial in 75 subjects with moderate plaque-induced gingivitis	3	14	↓PI, GI, PPD, BI with no significant difference between extract & powder	(116)
*Calendula officinalis* (1:3 concentration of tincture in water)	Mouthwash	Placebo-controlled, clinical trial in 240 subjects with gingivitis	3	180	↓PI,GI,SBI,OHIS	(45)
*Calendula officinalis *2%	Dentifrice	Double-blind, clinical trial in 40 subjects with established gingivitis-compared with placebo	4	28	↓PI, GI & BOP	(46)
*Camellia sinensis*	Sugar-free dragées	Placebo-controlled, double-blind clinical trial in 47 subjects	3	28	↓SBI, API	(55)
*Camellia sinensis* 5%	Mouthwash	Clinical trial in 30 subjects- compared with CHX	2	30	↓QHI, GBI, tooth stain, & tongue stain same in both groups ↓BI in both groups (test was better)	(57)
*Camellia sinensis *(catechins) 0.25%	Mouthwash	Single blind crossover clinical trial in 30 subjects- compared with CHX	3	15	↓PI same in both groups	(48)
*Camellia sinensis *(Green tea catechin) 1.0 mg/ml	Hydroxy-propyl-cellulose strips as a slow release local delivery system applied in pockets	Randomized, placebo-controlled clinical trial in 6 subjects with advanced periodontitis –group1 (scaling + tea) /group 2 (scaling + placebo) /group3 (tea only) /group 4 (placebo)	1	56	*In vitro* bactericidal effects against *Porphyromonas gingivalis, Prevotella intermedia, Prevotella nigrescens* ↓PPD only in group 1 Peptidase activity: ↓in group1 & ↑in group 2 No significant change in groups 3 & 4 Black-pigmented, Gram-negative anaerobic rods: ↓in group 1 & ↑in group 3 No significant change in group 2	(51)
*Camellia sinensis* 0.5% OR *Azadirachta indica* 2%	Mouthwash	Randomized, double-blind, clinical trial in 30 healthy subjects- compared with CHX	5	21	↓GI In all groups (better with herbal preparations) ↓PI, Maximum efficacy: 0.5% *C. sinensis* ↑OHIS, In all groups (better with herbal treatments preparations) ↑salivary pH, higher in herbal treatments	(124)
*Camellia sinensis *2%	Mouthwash	Randomized, placebo-controlled clinical trial in 110 subjects	5	28	↓GI, PI	(49)
*Camellia sinensis* 5%	Mouthwash	Single-blind, placebo-controlled, clinical trial in 50 subjects with chronic generalized plaque-induced gingivitis	1	35	↓PI, GI, PBI but not statistically significant	(53)
*Cinnamomum zeylanicum* 20%	Mouthwash	Randomized, triple-blind, controlled trial, a three-group parallel study in 105 subjects- compared with CHX	4	30	↓GI, QHI	(125)
*Copaifera *sp. 10%	Gel	Randomized placebo-controlled clinical trial in 23 subjects with experimental gingivitis	5	21	↓GBI, GI, PI compared with basline but no significant difference between groups	(126)
*Curcuma longa *10 mg/100 ml	Mouthwash	Clinical trial in 100 subjects- compared with CHX	3	21	↓GI & total microbial count: Same in both groups ↓QHI: in both groups (CHX was better)	(127)
*Curcuma longa* extract 1%	Gel	Uncontrolled pilot clinical trial in 10 subjects with severe gingivitis	1	21	↓PBI,GI	(63)
*Curcuma *sp. 0.1% + eugenol 0.01%	Mouthwash	Clinical trial in 60 subjects with mild to moderate gingivitis-compared with CHX	0	21	↓GI, PI, BAPNA Numerically but not statistically significant better than CHX	(64)
Curcumin (from *Curcuma longa*) 10 mg/ g	Gel	Randomized, double-blind clinical trial in 60 subjects- compared to CHX 10 mg & CHX-MTZ 10 mg	5	60	MGI, PI, BOP, PPD: no significant change ↓IL-1β & CCL28 levels in gingival crevicular fluid: test >CHX-MTZ >CHX	(61)
Curcumin (from* Curcuma longa)* 1%	Gel	Randomized clinical trial in 30 subject with severe gingivitis- compared with curcumin + SRP treatment	2	21	↓SBI, PI, GI Better in curcumin + SRP	(114)
*Cymbopogon* spp. oil 0.25%	Mouthwash	Randomized, double-blind, controlled parallel designed clinical trial in 60 subjects-compared with CHX	5	21	↓PI, GI and *Cymbopogon* was numerically but not statistically better than CHX	(128)
*Enteromorpha linza *1.5 mg/ ml	Mouthwash	Randomized, double-blind, controlled trial in 55 subjects – compared with Listerine	2	42	↓GI, QHI, GBI, bacterial strains (*Porphyromonas gingivalis, Prevotella intermedia*) Same in both groups	(129)
*Eucalyptus* *globulus* 0.6%- OR 0.4%-	Chewing gum	Randomized, double-blind, placebo-controlled, trial in 97 subjects with gingivitis	4	98	↓GI, PPD, BOP, plaque accumulation at both concentrations, No significant change in clinical attachment level	(115)
Eugenia uniflora 3%	Dentifrice	Randomized, double-blind, controlled clinical trial in 50 subjects–compared with fluoridated triclosan dentifrice	5	7	↓OHI only in control ↓GBI same in both groups	(130)
*Garcinia mangostana *	Gel	Controlled clinical trial in 31 subjects with periodontal pocket- compared with scaling only	2	90	↓BOP, PI, GI, PPD, clinical attachment, means percentage of *cocci*	(131)
*Glycyrrhiza glabra *30%	Mouthwash	Randomized, placebo-controlled clinical trial in 20 subjects	0	14	↓PI, GI	(132)
*Ilex rotunda *0.6%	Dentifrice	Randomized, double-blind, placebo-controlled clinical trial in 100 subjects-compared with normal dentifrice without active agents	5	84	↓GI,QHI	(133)
*Ixora coccinea* 0.2%	Mouthwash	Randomized, controlled clinical trial in 20 subjects- compared with CHX	2	28	↓GI, QHI, BOP: same in both groups ↑Lobenne tooth staining index in CHX	(134)
*Lactuca sativa* 200 mg nitrate	Daily consumption (systemic administration)	Randomized, double-blind, placebo-controlled clinical trial in 39 subjects with chronic gingivitis	5	14	No significant change in PCR ↓GI Lower in test ↑SNL higher in test	(135)
*Lippia sidoides* 1%	Mouthwash	Randomized, double-blind, parallel-armed pilot study in 55 subjects- compared with CHX	4	7	↓GI, PI, GBI No significant difference between test and CHX	(68)
*Lippia sidoides* 1%	Mouthwash	Randomized, double-blind, parallel-armed pilot study in 55 subjects- compared with CHX	4	7	↓GI, GBI, PI, salivary *Streptococcus mutans*	(67)
*Lippia sidoides* 10%	Gel	Parallel controlled clinical trial in 30 subjects- compared with CHX	5	90	↓PI, GBI same in both groups	(13)
*Lippia sidoides* 10%	Gel	Randomized, double-blind, placebo-controlled, crossover clinical trial in 22 subjects with experimental gingivitis	4	21	↓GI No significant change in GBI & PI	(66)
*Macleya cordata *0 .005% & *Prunella vulgaris *0.5%	Dentifrice	Double-blind, placebo-controlled, clinical trial in 40 subjects with gingivitis	2	84	↓PI ↓CPITN & PBI numerically but not statistically significant	(136)
*Magnolia officinalis *(magnolol 0.10% + honokiol 0.07%)	Chewing gum	Randomized, double-blind, controlled intervention trial in 117 subjects-compared with xylitol chewing gum or placebo chewing gum	5	30	Plaque pH Efficiency in maintaining the pH: Magnolia > Xylitol> Control No significant difference in BOP ↓Salivary *Streptococcus mutans *count	(117)
*Magnolia* *officinalis* 0.3%	Dentifrice	Randomized, double-blind, placebo-controlled clinical trial in 94 subjects	5	180	↓QHI,GI	(137)
*Matricaria chamomilla *1%	Mouthwash	Randomized, double-blind, placebo-controlled pilot study in 30 subjects- compared with CHX	4	15	↓VPI, GBI Same in CHX & Test	(74)
*Melaleuca* sp. 2.5%	Gel	Double-blind, longitudinal, non-crossover study in 49 subjects with severe chronic gingivitis-compared with CHX	3	56	GI: CHX>test > control PSS: CHX> control > test (not significant) PBI: Test> CHX> control	(56)
*Menthol* 18 mg %	Mouthwash	Double-blind, crossover, controlled clinical trial in 30 subjects-compared with CHX 0.2% & deionized water	1	5	↓PI, GI, GBI Less effective than CHX	(112)
*Ocimum gratissimum *	Mouthwash	Randomized, parallel, double-blind clinical trial in 30 subjects-compared with CHX	5	90	↓PI, GBI same in both groups	(138)
*Ocimum sanctum* 4%	Mouthwash	Randomized, triple blind, controlled trial in 108 subjects- compared with CHX	5	30	↓PI, GI same in both groups	(79)
*Polygonum aviculare* 1 mg/ml	Mouthwash	Uncontrolled clinical trial in 51 subjects with gingivitis	1	14	↑PI (however, the consistency of this plaque permitted its mechanical flushing easily) ↓GI	(139)
*Punica granatum * Or *Matricaria chammomila * Or *Salvadora persica*	Mouthwash	Randomized, double-blind, placebo-controlled clinical trial in 104 subjects with gingivitis	2	28	↓PI, Same in all groups ↓BOP Better in herbal groups Better taste and acceptability in *P. granatum*	(75)
*Punica granatum * 10%	Gel	Placebo-controlled, crossover, double-blind study in 23 subjects	4	21	No significant change in PI, GBI	(140)
*Punica granatum *6.25%	Mouthwash	Randomized, controlled, double-blind clinical trial in 35 subjects-compared with CHX	4	7, 12	↓GBI, PI only in CHX ↓saliva streptococci count in both groups (CHX was better )	(85)
*Punica granatum* 0.05%	Gel	Clinical trial in 40 subjects- group 1(mechanical debridement + test gel)/ group 2 (mechanical debridement + control gel)/ group 3 (test gel only)/ group 4 (control gel only)	2	21	↓PI, GI, PBI in groups 1 & 2 but not in groups 3 & 4 Significant difference at the end was seen only in PBI among groups Gram-negative *cocci *&* bacilli* (less in groups 1 & 3) & Gram-positive *cocci *&* bacilli* same in all groups Best results with group 1	(141)
*Punica granatum* 30%	Mouthwash	Randomized, single-blind, placebo-controlled clinical trial in 32 subjects with moderate gingivitis	1	28	↓Saliva total protein, AST activity & α-glucosidase activity ↑ceruloplasmin activity & radical scavenging capacity	(84)
*Punica granatum* 50-75 mg/ml	Mouthwash	Randomized, triple-blind, placebo-controlled clinical trial in 45 subjects- compared with CHX	5	4	↓GI with best effect by *P. granatum*	(83)
*Punica granatum *var *pleniflora *10 g in 240 ml	Mouthwash	Randomized, double-blind, clinical trial in 80 subjects with diabetes mellitus & gingivitis- compared with CHX	5	14	↓GBI, PPD, PI same in both groups ↓MGI better in test	(87)
*Rabdosia rubescens * 1: 960 mg of the herb +1000 mg simulating agent 2: 1000 mg of the herb + 960 mg simulating agent	Drop pill or tablets of simulation agent	Randomized, double-blind, double-simulation, positive-controlled parallel multi-center trial in 136 subjects with gingivitis	4	5	↓Major symptoms of Gingivitis 1 same as 2 ↓Minor symptoms of Gingivitis 1 same as 2 except in dry mouth and thirst which 1 was better than 1 Therapeutic effect 1>2	(142)
*Salvadora persica*	Miswak (chewing stick)	Randomized, single-blind, parallel-armed study in 30 subjects with mild to moderate gingivitis -group 1 (only toothbrush)/ group 2 (toothbrush+ Miswak)/ group 3 (only Miswak)	1	56	↓GI: Group 2 > group 1 = group 3 ↓QHI: group 2 > group 1 > group 3	(95)
*Salvadora persica*	Miswak (chewing stick)	Randomized, double-blind, controlled trial in 58 subjects with gingivitis	5	21	↓API & GI: No significant difference between groups composition of sub-gingival microbiota was same in both groups	(96)
*Salvadora persica *0.6%	Gum	Randomized, placebo-controlled clinical trial in 60 subjects with plaque induced moderate gingivitis- Either combined with SRP treatments or solely	4	14	↓GI, SBI No significant difference in PI	(91)
*Salvadora persica* 15 drops in 15 ml of water	Mouthwash	Randomized, placebo-controlled, clinical trial in 32 subjects with gingivitis-compared with CHX	1	14	↓CFU of plaque samples: CHX> *S. persica* No *in vitro* antibacterial effects	(94)
*Salvadora persica* 15 drops into 15 ml water	Mouthwash	Double-blind, placebo-controlled, crossover trial in 28 subjects	2	21	No significant change in PI ↓GBI ↓Salivary concentrations of *Streptococcus mutans*	(92)
*Salvadora persica* OR Parodontax OR Silca	Dentifrice	Controlled, clinical trial in 66 non-smoking subjects -compared with Colgate total	3	21	↓SBI: Parodontax = *S. persica *> colgate total ↓API: same in all groups	(93)
*S* *alvadora* * persica* ( 940 mg) + *Aloe vera*	Mouthwash	Randomized, double-blind controlled clinical trial in 76 patients under mechanical ventilation in ICU ward- compared with CHX	3	4	↓GI Better in test	(143)
*Schinus terebinthifolius *0.3125%	Mouthwash	Randomized, controlled, triple-blind, phase II clinical trial in 27 subjects with plaque induced gingivitis- compared with CHX	5	10	↓OHIS (amount of biofilm) only in CHX ↓GBI same in both groups	(15)
*Scutellaria baicalensis *0.5%	Dentifrice	Randomized, double-blind clinical trial in 40 subjects with experimental gingivitis-compared with fluoride toothpaste	5	21	PI, GI, VF%	(144)
*Streblus asper* 80 mg/ml	Mouthwash	Single-blind, crossover clinical study in 30 subjects-compared with distilled water	2	4	↓GI No significant change in PI, *Streptococcus mutans* count in plaque & saliva total salivary bacterial count	(145)
*Terminalia chebula* 10%	Mouthwash	Randomized, double-blind, controlled study in 78 subjects with gingivitis- compared with CHX	4	14	↓QHI, GI, pH same in both groups	(146)
*Terminalia chebula* 10%	Mouthwash	Randomized, double-blind, controlled trial in 60 subjects- compared with CHX	5	28	↓GI, PI Same in both groups	(101)
*Vaccinium myrtillus* (250 g (1) or 500 g (2))	Daily oral consumption	Placebo-controlled clinical trial in 24 subjects with gingivitis- compared with standard care (debridement)	2	7	↓BOP in standard care, 2 & placebo, but not 1 ↓IL-1β, IL-6, VEGF in gingival crevicular fluid only in group 2	(147)

GI: Loe & Silness gingival index; SBI: Muhlemann & Son's Sulcus bleeding index; PI: plaque index; PBI: papillary bleeding index; PHPI: Patient Hygiene Performance Index; CPITN: community periodontal index of treatment needs; BOP: Bleeding on probing; GBI: Gingival Bleeding index; VF%: biofilm vitality; API: approximal plaque index; OHIS: simplified Greene & Vermillion’s Oral Hygiene Index; BAPNA: The N-benzoyl-l-arginine-p-nitroanilide (BAPNA) assay used to analyze trypsin like activity of "red" complex microorganisms; CHX: chlorhexidine; QHI: Quigley & Hein plaque index; MGI: modified gingival index; PPD: probing pocket depth; MMP-8: matrix metalloproteinase-8;BA: biofilm accumulation; SAnB: anaerobic (SAnB) & aerobic (SAB) bacterial counts; NPI: Navy Plaque Index; PPI: planimetric plaque index; VPI: visible plaque index; PPBI: The periodontal probe bleeding index of Ainamo & Bay; PSS: plaque staining score; SBI: sulcus bleeding index; PSS: plaque staining score; LB: Lobene index; Parodontax: chamomile, echinacea, sage, rhatany, myrrh & peppermint oil; SRP: Scaling and Root Planing; PHP: patient hygiene performance; PMA: Proximal Marginal and attached gingival index; SNL: Salivary Nitrate Level; MTZ: metronidazole, IL: interleukin, IFN: interferon

**Table 2:** Clinical trials on the effectiveness of polyherbal formulations for the treatment of gingivitis

Arimedadi oil	Mouthwash	Clinical trial in 45 subjects with mild to moderate gingivitis- compared with CHX	3	21	↓GI, PI same in both groups	(148)
Essential oil mixture (thymol, eugenol and eucalyptus)	Dentifrice	Placebo-controlled double-blind, parallel, clinical study in 104 subjects	3	180	↓QHI,GI	(149)
Listerine	Mouthwash	Randomized, placebo-controlled, clinical trial in 32 subjects with gingivitis-compared with CHX	1	14	↓CFU of plaque samples: CHX> Listerine No *in vitro* antibacterial effects	(94)
Parodontax	Dentifrice	Randomized, double-blind clinical trial in 30 subjects-compared with standard fluoridated dentifrice	4	21	No significant change in QHI ↓GI only in test	(150)
Polyherbal preparation	Dentifrice	Randomized, double-blind, placebo-controlled clinical trial in 66 subjects	5	168	↓BOP, PPD, SAnB, QHI	(105)
Polyherbal preparation	Dentifrice	Randomized, double-blind, placebo-controlled clinical trial in 60 subjects	4	84	↓GI, BOP, SAnB	(106)
Polyherbal preparation	Gel & powder	Randomized, double-blind, placebo-controlled, clinical trial in 113 subjects with chronic generalized gingivitis -compared with CHX	5	168	↓GI, PI, microbial count same in test groups and CHX No significant difference between gel & powder	(151)
Polyherbal preparation	Mouthwash	A Randomized, double-blind, placebo-controlled clinical trial in 17 subjects with gingivitis	5	84	No significant difference in PI, GI & relative abundance of two periodontal pathogens	(152)
Polyherbal preparation	Mouthwash	Randomized, double-blind, placebo-controlled trial in 60 subjects with gingivitis-compared with CHX	3	14	↓QHI, MGI, GBI Same in herbal test and CHX	(9)
Polyherbal preparation	Mouthwash	Clinical study Phase I in 30 subjects with periodontitis Phase II in 34 subjects with gingivitis- compared with CHX	1	Phase I: 28, Phase II: 14	Phase I ↓PPD, BOP, clinical attachment Efficiency (numerically but not statistically significant): CHX>test>placebo Phase II ↓GI, BOP (numerically but not statistically significant): test = CHX > placebo ↓PI: same in all 3 groups	(108)
Polyherbal preparation	Mouthwash + sub-gingival irrigator	Randomized, double-blind clinical study in 89 subjects-group1(irrigator + test mouthwash)/group 2 (irrigator+ conventional mouthwash)/group 3(conventional mouthwash only)	3	90	↓GI in group 2, group 1, but not in group 3 ↓SBI only in group 1 ↓PI in all groups No significant change in PPD	(153)
Polyherbal preparation	Transmucosal herbal periodontal patch	Randomized, single-center, double-blind placebo-controlled, crossover, longitudinal phase II trial in 50 subjects with gingivitis	3	15	↓GI ↓gingival crevicular fluid β-glucuronidase enzymatic activity	(102)
Triphala (*P. emblica*, *T. bellirica*, and *T. chebula*) 10 g in 10 ml water	Mouthwash	Randomized ,double-blind, multicenter clinical trial in 120 hospitalized periodontal disease subjects-compared with CHX	5	15	↓PI, GI same in both groups	(98)
Triphala 0.6% (*P. emblica*, *T. bellirica*, and *T. chebula*)	Mouthwash	Controlled clinical trial in 1431 healthy subjects -compared with CHX and placebo	3	270	↓PI, GI, *Streptococcus* count in both groups ↓*Lactobacilli* count more pronounced in test group	(97)
Triphala 10% (*P. emblica*, *T. bellirica*, and *T. chebula*)	Mouthwash	Randomized, double-blind, crossover study in 120 healthy subjects- compared with CHX	5	30	↓QHI, GI same in both groups	(154)

GI: Loe & Silness gingival index; SBI: Muhlemann & Son's Sulcus bleeding index; PI: plaque index; PBI: papillary bleeding index; CHX: chlorhexidine; QHI: Quigley & Hein plaque index; PPD: probing pocket depth; SAnB: anaerobic bacterial count

***Polyherbal preparations***

*Triphala*

Triphala is a traditional multi-component herbal preparation containing three main ingredients, *Terminalia bellirica* (Gaertn.) Roxb., *Terminalia chebula* (Gaertn.) Retz., and *Phyllanthus emblica* L. (Synonym: *Emblica officinalis*). Triphala mouthwash showed an effectiveness similar to CHX according to PI, GI and *Streptococcus* count reduction rate; however, triphala had a more pronounced effect on *Lactobacillus* count (97). Triphala was also compared with CHX in another study on 120 hospitalized periodontal disease subjects and was equally effective in reducing PI and GI (98). Same results were obtained in another study where triphala and CHX were compared in reducing QHI and GI (99). In addition, *T. chebula* which is an ingredient of triphala was individually evaluated in two trials (Table 1). In a clinical trial, *T. chebula* mouthwash was able to neutralize salivary pH. It also decreased QHI and GI indices similarly to CHX without any taste alteration and discoloration (100). In another study in 60 subjects, *T. chebula* mouthwash reduced PI and GI and the effectiveness was equal to CHX (101).

*Miscellaneous polyherbal preparations*

A transmucosal herbal periodontal patch containing a mixture of herbs including *Centella asiatica* (gotu kola), *Echinacea purpurea*, and *Sambucus nigra* (elderberry) was clinically effective in reducing GI and gingival crevicular fluid β-glucuronidase (BG) enzymatic activity (135). The GCF BG level reflects the quantity of polymorphonuclear leukocytes found in the sulcus and may be a more accurate assessment of inflammation found in the periodontal sulcus than subjective clinical signs of inflammation (136). HM-302 is a mixture of the same herbs used to treat gingivitis in a study (Table 2). Its effect was compared to Listerine, cetylpyridinum chloride or water. PI, GI and BOP deteriorated in all groups except HM-302 (137).

A Sri-Lanka polyherbal preparation containing *Acacia chundra*, *Adhatoda vasica*, *Mimusops elengi*, *Piper nigrum*, *Pongamia pinnata*, *Quercus infectoria*, *Syzygium aromaticum*, *Terminalia chebula*, and *Zingiber officinale* significantly improved QHI, PPD, BOP indices, as well as the salivary aerobic and non-aerobic bacterial counts (138). In another study in 60 subjects, same preparation reduced GI, BOP and salivary aerobic and non-aerobic bacterial counts (139).

Another polyherbal preparation containing hydroalcoholic extracts of *Zingiber officinale*, *Rosmarinus officinalis*, and *Calendula officinalis* was evaluated in 60 subjects. MGI, GBI and QHI indices were improved to the same levels as CHX (9).

Rinsing with a polyherbal mouthwash (*Salvia officinalis, Mentha piperita, menthol, Matricaria chamomilla, Commiphora myrrha, Carvum carvi, Eugenia caryophyllus *and *Echinacea purpura*) accompanied by a sub-gingival irrigator had significant effect on PI, GI and SBI indices (148).

Radvar *et al.* prepared a polyherbal mouthwash using *Salix alba, Malva sylvestris*, and* Althaea officinalis *herbs which was evaluated in subjects with either gingivitis or periodontitis (Table 2). CHX or test mouthwash were not significantly different than placebo control in improving PPD, BOP and clinical attachment in those with periodontitis; however, they successfully reduced GI and BOP (145).

A dentifrice containing gotu kola and magnolia was compared with conventional toothpastes and decreased PHP, PMA and malodor indices after 14 days (149). PMA index is an easy method to help figure out the inflammatory portion from the normal portion at the divided areas by comparing each side as papillary, marginal and attached gingiva (150). PHP is a simplified patient hygiene performance evaluation.

***Phytochemicals***

*Menthol*

Menthol is a monoterpene which is found in different types of mint, as well as several other plants of the Lamiaceae family. The compound is widely used in food industries as a natural flavoring agent, and is also a main part of several oral health products like dentifrices, chewing gums, and mouthwashes (151). A solution of menthol showed less effectiveness in reducing PI, GI and GBI as compared to CHX in 30 subjects in a clinical trial (124).

*Curcumin*

Curcumin is a secondary metabolite with diarylheptanoid structure which is mainly extracted from the rhizome of turmeric (*Curcuma longa*) and has shown significant biological activities like antioxidant, anti-inflammatory, and cytoprotective effects (152). Curcumin massaged on gingiva in addition to SRP treatments significantly reduced GI, PI and SBI indices compared to baseline (111).

## Discussion

Herbal elements are gaining attention as both preventive plaque control approaches and as adjunctive treatments. Among single herbal preparations, many studies have focused on *Aloe vera* (aloe), *Punica granatum* (pomegranate), *Salvadora persica* (miswak) and *Camellia sinensis* (tea). Polyherbal mixtures have also been studied regarding their effect on the reduction of microbial count and plaque index and other measures. Triphala, for instance is a mouth rinse composed of *T. bellirica*, *T. chebula*, and *P. emblica* which showed positive effects similar to that of CHX (97-99). 

Plant secondary metabolites including menthol from mint species and curcumin from turmeric also showed considerable therapeutic activity for the management of gingivitis-induced inflammation, bleeding, and plaque formation (61, 111, 124).

There were a wide diversity of dosage forms and formulations in different studies (Figure 1). Mouthwashes and dentifrices were the most popular forms of administration (Table 1). Green tea has been administered as various dosage forms such as mouth rinse, candies and slow release local delivery systems (39, 51, 55). Some plants like eucalyptus (114), frankincense (106), miswak (91), and magnolia (122) were prepared as chewing gum which might be a favorable dosage form specially for youngsters. Some other extracts such as turmeric (63) and barberry (105) were formulated as gels which, considering the safety of the plant used, can be applied to the damaged areas and would be of great interest in children who might have poor degree of cooperation in using mouthwashes or dentifrices. 

The main mechanisms by which herbal elements improve the condition of periodontium are described in Figure 1. Immediate bleeding is a result of inflammation in gummy tissues. A combination of host susceptibility and microbial accumulation in form of plaque culminates in inflammation. One of the main mechanisms of medicinal plant to control gingivitis is their anti-inflammatory activity. Some medicinal plants such as pomegranate, tea, and chamomile are rich sources of flavonoids and tannins which are potent anti-inflammatory and astringent phytochemicals and thus, can control both bleeding and inflammation. Aside from different bleeding and inflammation indices reduced during the studies (Table 1), some trials have measured the crevicular level of inflammation biomarkers which strongly support the anti-inflammatory activity of herbal drugs (61). 

Another important effect is to control the microflora of oral cavity. Several studies have demonstrated the positive role of herbal extracts to reduce the bacterial count of oral pathogens and plaque formation (Table 1). Rinsing with herbal mouth washes or applying herbal dentifrices, as well as all other sorts of application, can show bactericidal effect and counteract bacterial metabolism (153, 154).

Also, some studies assessed the effect of a combination of herbal treatments along with conventional mechanical dental practices such as scaling (50) which showed a synergistic effect; suggesting that herbal products can be used as a complementary therapy to improve the effectiveness of conventional therapies (25). 

## Conclusion

Taken together, this paper supports the efficacy of several medicinal plants for the management of gingivitis based on the current clinical evidence; however, available clinical data has several limitations such as short course of study, Small sample size, and lack of blinding which remains the effectiveness of some preparations to be unclear. Thus, future well-designed clinical studies are essential in case of several medicinal plants for their efficacy to be confirmed in gingivitis. 
